# Flexible Moisture–Electric Generator Based on Vertically Graded GO–rGO/Ag Films

**DOI:** 10.3390/ma18122766

**Published:** 2025-06-12

**Authors:** Shujun Wang, Geng Li, Jiayue Wen, Jiayun Feng, He Zhang, Yanhong Tian

**Affiliations:** 1 State Key Laboratory of Precision Welding & Joining of Materials and Structures, Harbin Institute of Technology, Harbin 150001, China; 2 Zhengzhou Research Institute, Harbin Institute of Technology, Zhengzhou 450041, China

**Keywords:** nanogenerators, moisture–electricity generators, graphene oxide, inkjet printing

## Abstract

Moisture–electricity generators (MEGs) hold great promise for green energy conversion. However, existing devices focus on the need for complex gradient distribution treatments and the improvement in output voltage, overlooking the important role of the graphene oxide (GO) oxidation degree and the response time and recovery time in practical application. In this work, we develop printed MEGs by synthesizing reduced graphene oxide/silver nanoparticle (rGO/Ag) composites and controlling the GO oxidation degree. The rGO/Ag layer serves as a functional component that enhances cycling stability and shortens the recovery time. Additionally, compared to conventional rigid-structure devices, these flexible MEGs can be produced by inkjet printing and drop-casting techniques. A 1 cm^2^ MEG can generate a voltage of up to 60 mV within 2.4 s. Notably, higher output voltages can be easily achieved by connecting multiple MEG units in series, with 10 units producing 200 mV even under low relative humidity (RH). This work presents a low-cost, highly flexible, lightweight, and scalable power generator, paving the way for broader applications of GO and further advancement of MEG technology in wearable electronics, respiratory monitoring, and Internet of Things applications.

## 1. Introduction

As the increasing worldwide need for energy continues to rise, the creation of sustainable, environmentally friendly, and affordable power generation technologies has become a critical priority [1]. Self-powered energy has emerged as a promising technology, offering reliable power generation from ambient environmental sources. At present, self-powered energy technologies include solar cells, thermoelectric generators [2], triboelectric nanogenerators [3], piezoelectric generators [4] and moisture–electricity generators (MEGs) [5]. Among these technologies, MEGs have attracted significant attention due to the ubiquity of water and their independence from geographic and climatic conditions. The adsorption of water molecules by hygroscopic materials triggers the dissociation of surface functional groups, releasing charged ions that generate an output voltage through electrochemical interactions [6,7,8]. Recently, various materials have been used for moisture-enabled power generation, including carbon materials [6,9,10], polymers [11,12,13], biomaterials [14,15,16], and semiconductor materials [17,18,19]. Graphene oxide (GO), a representative two-dimensional material [20,21,22,23,24,25,26,27,28,29,30], has shown great potential for moisture-enabled power generation since the first report of GO-based materials in this field [31]. Because of its well-established fabrication techniques, high sensitivity to water molecules, large particular surface area, and availability of functional groups, GO presents significant prospects for the development of moisture-enabled energy devices [32,33]. Although GO-based MEGs demonstrate promising electrical output [6,34,35], they still suffer from limited cycling stability and complex fabrication processes. According to the working mechanism of MEGs, ion gradients are a critical factor in facilitating power generation [36,37]. Several strategies have been developed to achieve functional group gradients within materials, including electrochemical treatment [31,38,39], thermal annealing [40], laser reduction [38,41], and UV photoreduction [42,43]. Chen [42] employed UV irradiation treatment to produce a sandwich-like layer constructed of rGO-GO-rGO. The generator had the ability to output 215.7 mV in 1 h at 85% humidity. Cheng [40] presented a directional thermal strategy for GO assembly. The response time required 100 s even if the device’s output voltage was excellent. GO was employed in MEGs for the first time, according to Xu et al. [31]. In 3 s, the device generated an output voltage of 20 mV. Despite the fact that their work shows the promise for green energy conversion, the technical requirements for complex processing methods and costly equipment further complicate fabrication and raise production costs, which ultimately impede a wider application and further exploration of GO materials in MEG systems. In gas-sensing applications, reduced graphene oxide/silver (rGO/Ag) is a promising gas-sensing material demonstrating good sensing capabilities. The surface decoration of silver nanoparticles enhances the sensing capability and promotes gas molecule adsorption [44].

Owing to their exceptional sensitivity to humidity, MEGs have been increasingly applied in recent years to applications such as respiratory monitoring [43,45] and non-contact sensing [38,46]. However, traditional GO-based MEGs typically rely on rigid structures, which present significant challenges in terms of miniaturization, mechanical flexibility, weight reduction, and scalability. These limitations hinder their widespread integration into wearable or portable electronics. To overcome these issues and achieve durable, sensitive performance, the development of flexible device architectures is essential. Printed electronics offers a promising approach, employing various printing techniques to deposit functional inks onto substrates, followed by post-processing treatments to ensure strong adhesion between functional layers and substrates. This strategy enables the scalable fabrication of diverse electronic devices [47]. Common printing methodologies include screen printing [48,49], 3D printing [50,51,52,53,54,55,56,57,58,59,60], inkjet printing [61], electrohydrodynamic printing [62,63], and aerosol jet printing [64]. In early works, Liang et al. [65] fabricated a GO-based MEG via screen printing, while He et al. [66] employed analogous screen-printing techniques to develop a PSSA-PDDA planar MEG. Compared to screen printing, inkjet printing as an additive manufacturing method offers superior material compatibility, high resolution, excellent conformal integration, and cost-effectiveness.

Therefore, we aim to develop a moisture-enabled electricity generation which can respond rapidly and manufacture easily. In this study, we present a rapid-response MEG based on a GO and rGO/Ag layer featuring facile manufacturability, low cost, mechanical flexibility, printability, and scalability. GO was selected for its tunable oxygen-containing functional groups, while rGO/Ag was incorporated for superior adhesion capability. By using inkjet printing and drop-casting techniques, the device was fabricated on flexible polyethylene terephthalate (PET) substrates. The sensing layer, consisting of GO with a high density of hydrophilic hydroxyl groups, and the transition layer, formed by rGO/Ag nanocomposites, work synergistically to enable a 1 cm^2^ device to generate an output voltage of 60 mV within 2.4 s. Notably, in contrast to conventional gradient fabrication methods, we developed a simplified gradient construction strategy that significantly streamlines the manufacturing process while maintaining ion gradient. This voltage generation mechanism arises from the gradient distribution of GO induced by gravitational sedimentation during the drop-casting process. The incorporation of the rGO/Ag transition layer significantly enhanced the cyclability of the device and reduced the recovery time. Furthermore, the scalable inkjet-printed arrays achieved a 200 mV output in low-relative-humidity (RH) conditions. This work introduces a novel strategy for the scalable integration of MEGs, demonstrating potential applications in wearable electronics and environmental sensing through its unique combination of conformal fabrication and rapid response.

## 2. Experimental

### 2.1. Materials and Equipment

Graphite powders (300 meshes and 10,000 meshes, 0.5 μm–7 μm) were purchased from Nanjing Greifa Carbon Material Co., Ltd., Nanjing, China. Potassium permanganate (KMnO_4_), polyvinylpyrrolidone (PVP, k90), silver nitrate (AgNO_3_), sulfuric acid (H_2_SO_4_), hydrochloric acid (HCl), glucose (C_6_H_12_O_6_), sodium hydroxide (NaOH), and hydrogen peroxide (H_2_O_2_) were provided by Macklin Biochemical Co., Ltd., Shanghai, China. Silver nanowires (AgNWs, D = 60–90 nm, L = 40–70 μm, 1 wt%) were obtained from Zhejiang Kechuang Advanced Materials Co., Ltd., Hangzhou, China. Inkjet printing equipment was purchased from Hubei Huaweike Intelligent Co., Ltd., Ezhou, China. The humidity experimental setup containing a Constant Humidity Sealed Chamber, saturated moisture flowmeter, and commercial hygrograph was bought from Alibaba, China. All the materials were used as received without further purification.

### 2.2. Synthesis of GO

**Figure 1:**
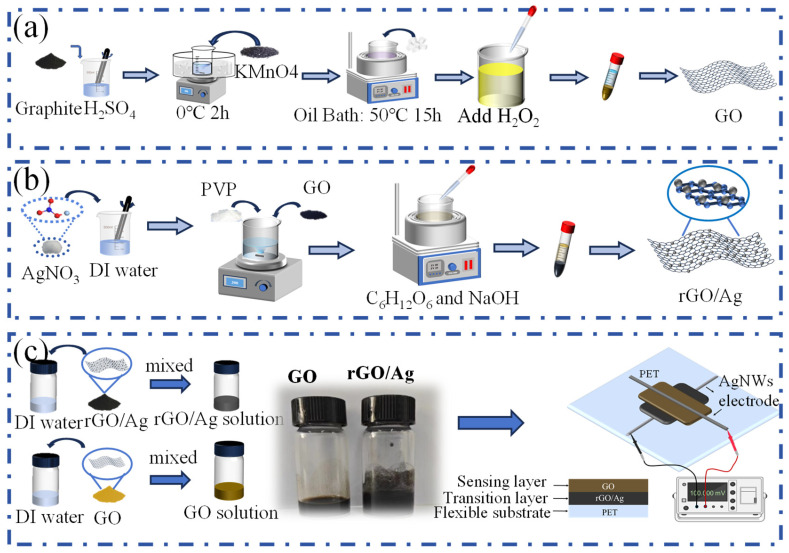
Fabrication process of GO, rGO/Ag, and the MEG device. (**a**) Diagrammatic illustration of the GO preparation procedure. (**b**) Schematic illustration of rGO/Ag synthesis via liquid-phase reduction. (**c**) Preparation of GO and rGO/Ag functional inks and schematic diagram of the MEG device.

Here, we prepared GO by the modified Hummers method [67]. Figure 1a shows the synthesis process. Briefly, graphite powder (1 g, 300 meshes) was added into 50 mL H_2_SO_4_ with stirring at 300 rpm. Next, while maintaining the temperature below 20 °C, the previously mentioned solution was gradually mixed with 3 g of KMnO_4_ while being stirred at 500 rpm. After being moved to the oil bath at 50 °C, the mixture was agitated for 15 h at 500 rpm. In the process, ice cubes were crushed and then gradually added to the mixture.

The 30% H_2_O_2_ solution was added to the solution to further treat it. HCl and deionized (DI) water were employed to clean the solution several times until it was neutral. Finally, solid GO was obtained by freeze-drying for 3 days. Notably, GO prepared by this method was used to synthesize rGO/Ag.

### 2.3. GO Synthesis for MEGs with Different Oxidation Levels

According to a modified Hummers method [68], GO with different oxidation levels were prepared. In short, 50 mL of H_2_SO_4_ was mixed with 1 g of graphite powder (1:10,000 mesh) while stirring the mixture at 300 rpm. Then, KMnO_4_ was gradually dissolved in the mixture in order to keep the temperature under 20 °C. After being transferred to an oil bath at 40 °C, the mixture was agitated for an hour. After that, the reaction system was heated to 95 °C and kept there for another 15 min after 50 mL of water was supplied. The solution was gradually poured into 90 mL of water once the temperature had dropped to 60 °C. To further treat the solution, 30% H_2_O_2_ was added. After that, the solution was regularly cleaned with DI water and HCl several times. Finally, solid GO was obtained by freeze-drying the solution for three days. Table 1 demonstrates the reaction conditions along with the matching GO samples.

### 2.4. Synthesis of rGO/Ag

The preparation procedure of rGO/Ag is shown in Figure 1b [69]. In the absence of light, AgNO_3_ was dissolved in DI water to create an AgNO_3_ solution at 0.1mol/L. After that, 170 mg PVP and 34 mg GO were gradually added into the solution. The solution was heated to 80 °C for 10 min at 800 rpm in an oil bath. After that, the entire system was kept at this temperature without agitation for 2 h. The solution was then progressively supplied with 30 mL of glucose (0.15 mol/L) and sodium hydroxide (1 mol/L) while stirring for 1.5 h. After that, the mixture was cleaned three times using anhydrous ethanol and DI water. Finally, solid rGO/Ag was obtained by freeze-drying the solution for 3 days.

### 2.5. The Design and Assembly of the MEG

As shown in Figure 1c, the functional ink consisted of GO and rGO/Ag. The GO ink (5 mg/mL) and rGO/Ag ink (5 mg/mL) were prepared by dispersing the respective powders in deionized (DI) water. The MEG functional unit featured a sandwich-like structure composed of a flexible PET substrate, an rGO/Ag transition layer, a GO sensing layer, and two AgNWs electrodes by printing. The PET sheet served as the substrate for the MEG. The bottom electrodes of the device were created by printing AgNWs onto the PET surface. After drying, the functional film was fabricated by a simple drip-coating process: the homogeneous ink solution was deposited onto the Ag electrode using a 20 μL pipette and then dried [65]. The complete functional film consisted of six layers, with the bottom three layers serving as the rGO/Ag transition layer and the top three layers forming the GO sensing layer. This straightforward fabrication process avoids the need for complicated chemical treatments.

### 2.6. Characterization

Atomic force microscopy (AFM, Dimension Fastscan, Bruker, Billerica, MA, USA) and scanning electron microscopy (SEM, TESCAN AMBER, TESCAN, Brno, Czech Republic) fitted with energy-dispersive X-ray spectroscopy (EDS) were used to characterize the microstructures of GO and rGO/Ag.

The SEM used in this study achieves a maximum magnification of 200,000×, providing sufficient resolution. The sample preparation methods for SEM and AFM analyses were as follows: Freeze-dried GO and rGO/Ag were separately uniformly dispersed in DI water via 30 min ultrasonic treatment to form solutions with a concentration of 0.5 mg/mL. Each solution was then drop-cast onto silicon wafers and dried under ambient conditions. The chemical structure evolution of GO and rGO/Ag was investigated using X-ray photoelectron spectroscopy (XPS, ESCALAB 250Xi, Thermo Fisher Scientific, Waltham, MA, USA) and Raman spectroscopy (inVia-Reflex). A monochromatic Al Kα X-ray source (1486.6 eV) was used for the XPS examination. High-resolution spectra were calibrated based on the carbon C 1s peak at 284.8 eV. Deconvoluted C1s and O1s spectra were used to characterize the chemical states, whereas wide-scan survey spectra of pure GO and rGO/Ag composites were used to evaluate the surface elemental composition. The Raman spectroscopy analyses were conducted using a 532 nm diode laser with a spot size of 1 μm^2^ at room temperature. GO and rGO/Ag samples were freeze-dried and ground into powder in order to characterize them for Raman spectroscopy and XPS.

A Keysight 34465A (Santa Rosa, CA, USA) digital multimeter was used to measure the electrical output performance of the MEG devices under various humidity levels. We created a humidity cabinet (Figure S4) that can precisely change humidity via the flow rate of dry N_2_ and wet N_2_ in order to properly evaluate the electrical performance of the MEGs. Environmental parameters were kept at 25.0 ± 0.5 °C and 30 ± 3% relative humidity throughout the test. Humidity was measured using a commercial temperature/humidity sensor. The humidity cabinet was adjusted to the desired relative humidity levels before the electrical output performance test. The output voltage was continuously observed until it stabilized. After that, the device was moved immediately to a drying chamber with dry N_2_ until the voltage reached a stable level. To guarantee measurement repeatability, this procedure was carried out three times in a row for each device.

## 3. Results and Discussion

### 3.1. GO Structural Analysis

**Figure 2:**
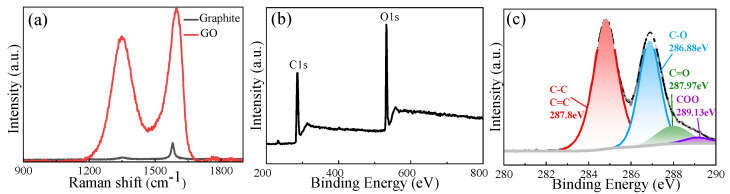
Structural and chemical characterization of graphene oxide (GO). (**a**) Raman spectra of graphite and GO; (**b**) XPS survey scan spectra; (**c**) C1s XPS spectrum of GO.

According to the microstructural investigation, the GO had a sheet-like structure with surface wrinkles and scrolled edge configurations (Figure S1a,b). Cross-sectional characterization confirmed a multilayer architecture resulting from interlayer stacking of graphene sheets. The AFM image in Figure S1c,d demonstrated individual sheet thicknesses reaching 4 nm, indicating that the obtained GO is a multilayer structure. As shown in Figure 2a, distinct spectral differences are observed between the two materials in the Raman spectra. Two distinctive peaks for GO samples may be seen at 1300 cm^−1^ and 1500 cm^−1^. While the D-band in 1300 cm^−1^ results from structural flaws that trigger out-of-plane vibrational modes, the G-band in 1500 cm^−1^ is associated with the vibrational mode of sp^2^ hybrid structures [70]. The intensity ratio (I_D_/I_G_) of these bands quantitatively reflects the defect density in graphene-based materials. In contrast to graphite, which has a strong G band at 1579 cm^−1^, GO has a widened and displaced G band at 1596 cm^−1^ and an obvious D band with I_D_/I_G_ = 1.29. This significant increase in the I_D_/I_G_ ratio compared to pristine graphite confirms the partial conversion of sp^2^ hybridized carbon domains to sp^3^ configurations during oxidation, accompanied by the formation of oxygen-containing functional groups. XPS was employed to quantitatively analyze the surface composition and chemical states. The C1s spectrum of GO (Figure 2b) deconvolutes into four characteristic components [71]. As demonstrated in Figure 2b, there is a C1s peak and a stronger O1s peak. The high quality of the produced GO is demonstrated by the lack of extraneous peaks, and successful oxidation is confirmed by the C/O atomic ratio of 2.3. A C=C/C-C band, which occurs at 284.5 eV, a C-O band stretching at 286.88 eV, a C=O band stretching at 287.97 eV, and a COO peak extending at 289.13 eV are all seen in Figure 2c. Table S1 provides a summary of the quantitative distribution of these oxygen functionalities, illustrating the various kinds of oxygen functional groups present in GO throughout the oxidation process.

### 3.2. rGO/Ag Structural Analysis

**Figure 3:**
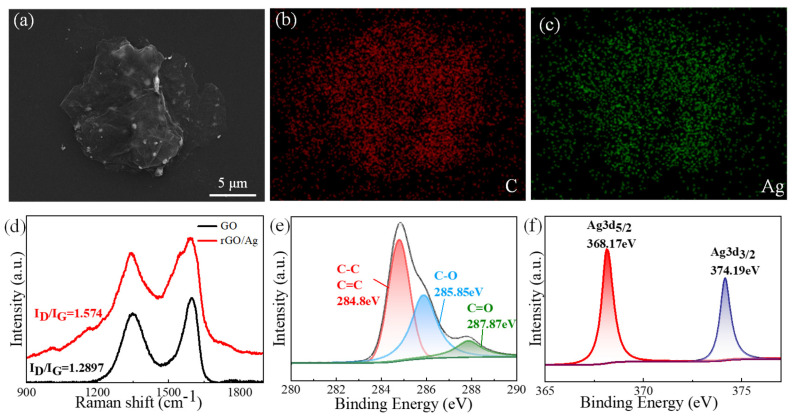
Characterization of rGO/Ag. (**a**) SEM image of rGO/Ag; (**b**,**c**) EDS elemental mapping images of rGO/Ag; (**d**) Raman spectra of GO and rGO/Ag; (**e**) C 1s XPS spectra of rGO/Ag; (**f**) Ag 3d XPS spectra of rGO/Ag.

Figure 3a displays a SEM image of the rGO/Ag, which reveals its structure. The rGO/Ag sheets exist as few-layer or monolayer sheets. Additionally, EDS images of rGO/Ag are shown in Figure 3b,c. The Ag element is evenly distributed throughout the rGO sheets, confirming the distribution of Ag nanoparticles (AgNPs) across the rGO matrix. Figure 3d shows the Raman spectra of rGO/Ag and GO, demonstrating characteristic D and G bands for both materials. Compared to GO, the intensity of the D peak and G peak are enhanced due to the surface-enhanced Raman scattering (SERS) of AgNPs [72]. In relation to the G band, the data show an increase in D band intensity, which corresponds with the restoration of the sp^2^ structural domains. During the conversion of GO to rGO/Ag, the I_D_/I_G_ ratio increases from 1.29 (GO) to 1.57 (rGO/Ag), indicating the formation of new and isolated smaller graphitic domains [73]. As seen in Figure S2, the results confirm the high quality of rGO/Ag via the existence of C1s, O1s, and Ag3d elements, as well as the lack of other peaks. The result of the XPS spectra indicates the presence of C1s, O1s, and Ag3d elements and the absence of additional peaks, confirming the high purity of the rGO/Ag. Similar to GO, C1s spectra (Figure 3e) can be divided into three types of carbon functional groups. The Ag3d spectra in Figure 3f exhibit distinct peaks for Ag3d_5/2_ and Ag3d_3/2_, confirming the presence of Ag with high purity [74]. Table 2 compares the oxidation levels of GO and rGO/Ag. Compared to GO, the C/O ratio of rGO/Ag increases to 7.56. The fact that the O1s ratio of rGO/Ag is substantially lower than that of GO indicates that GO is effectively restored by glucose.

### 3.3. Structural Analysis of GO with Different Oxidation Degrees

**Figure 4:**
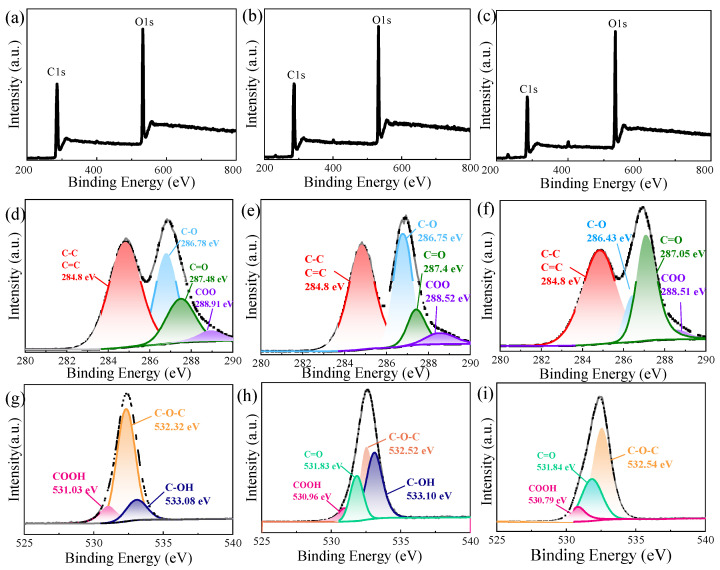
XPS spectra of GO-1,GO-2 and GO-3. (**a**) Survey spectra of GO-1; (**b**) survey spectra of GO-2; (**c**) survey spectra of GO-3; (**d**) C1s spectra of GO-1; (**e**) C1s spectra of GO-2; (**f**) C1s spectra of GO-3; (**g**) O1s spectra of GO-1; (**h**) O1s spectra of GO-2; (**i**) O1s spectra of GO-3.

SEM images of GO-1, GO-2, and GO-3 at different magnifications are presented in Figure S3. Gently and mildly oxidized materials such as GO-1 and GO-2 have larger sheets; however, when oxidation levels rise, the sheet size significantly decreases. The smallest sheet size is observed in GO-3, with a lateral dimension of approximately 6 μm (Figure S3e). The XPS spectra of GO samples are shown in Figure 4a–c. As the stirring speed and the amount of KMnO_4_ increase, the intensity of the O1s peak gradually increases relative to the C1s peak. As summarized in Table 3, the C/O atomic ratios for GO-1, GO-2, and GO-3 are 2.33, 2.14, and 1.76, respectively, indicating that the oxidation degree of GO can be effectively enhanced by increasing both the stirring speed and the oxidant concentration.

As the degrees of oxidation increase, XPS spectra show a clear development of the oxygen functional groups. Figure 4d illustrates that GO-1 has C=C bonds (284.8 eV), as well as COO (288.91 eV), C=O (287.48 eV), and C-O (286.78 eV). In comparison to GO-1, progressive oxidation in GO-2 is represented by a rise in C-O and a decrease in C=C (Figure 4e). At maximum oxidation (GO-3, Figure 4f), the C=O group increases while C-O groups decline, indicating that by changing the reaction conditions of GO preparation, not only can the oxidation degree of GO be changed, but the types of oxygen-containing groups can also be tuned.

In Table 3, we further analyzed the change in GO oxygen-containing group content under different oxidation levels. Compared to GO-1, the number of C-O groups in GO-2 increases to 49.29%. According to the XPS analysis of GO-3, raising the oxidation level further induced the formation of C=O (34.28%) at the expense of C-O (12.58%).

As established in prior works [71,75,76], C=O and COO bonds preferentially populate edge sites and form basal plane defects. This makes it abundantly evident that C-O suffers additional oxidation to generate C=O, leading to a smaller size, as shown in the SEM figures. In terms of O1s spectra, the corresponding peaks of C-OH,C=O,C-O-C,COOH of GO-2 (Figure 4h) appear at 533.1 eV, 531.83 eV, 532.52 eV and 530.96 eV, respectively [71]. Meanwhile, for the GO-1 and GO-3 samples, three main peaks are observed in Figure 4g,i. Table S2 shows that GO-2 samples have the most hydroxyl groups. Overall, the combined XPS and SEM data show that COOH and C-OH groups are mostly generated at gentle and lower oxidation levels, and that as the oxidation level rises, they are progressively transformed into C=O and C-O-C groups.

### 3.4. Output Performance of MEGs Under Moisture

**Figure 5:**
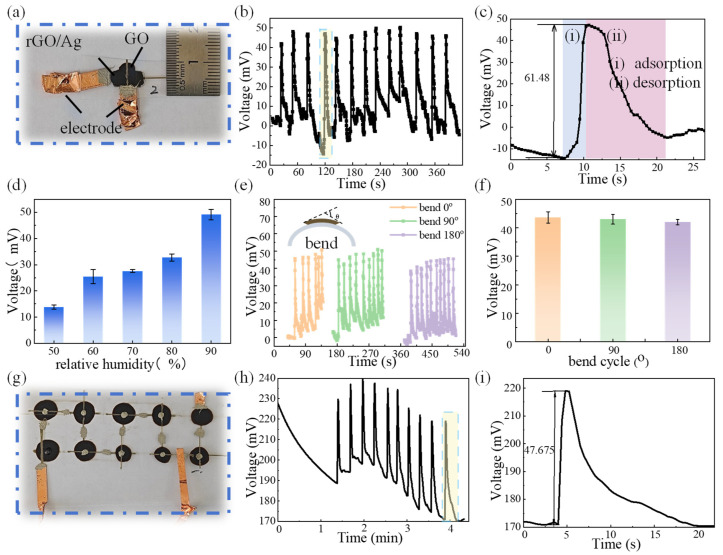
The MEG’s electrical output performance. (**a**) Optical image of one MEG unit; (**b**) output voltage cycles by a single MEG at ΔRH = 80%; (**c**) output voltage cycles of a single MEG unit at ΔRH = 80%; (**d**) voltage outputs of a single MEG under different humidity levels; (**e**,**f**) output voltage at different bending angles; (**g**) optical image of a printed MEG array; (**h**) voltage output of 10 MEG units connected in series under 40% RH; (**i**) output voltage of the arrays under ΔRH = 30%.

As the functional material of MEGs, GO is rich in hydrophilic -OH and -COOH groups, providing ample active sites while ensuring outstanding moisture adsorption and desorption capabilities [77,78]. GO-2 is chosen as the optimal functional material for MEGs due to its abundant oxygen-containing groups (-OH and -COOH). As shown in Figure 5a, a printed MEG unit with an area of only 1 cm^2^ was for the electrical output test. The environmental humidity was controlled using moisture-carrying nitrogen (N_2_) gas (Figure S4). When the device is stored in drying cabinet, there is no voltage generated. When the device is exposed to moisture (ΔRH = 80%) and inserted in a test circuit, a rapid voltage increase is observed. Subsequently, when the MEG is stored in drying cabinet, the voltage decreases to the original state, indicating that a full cycle is finished. After many cycles, the output voltage remains consistent with the initial output, which means high stability and reproducibility (Figure 5b). As shown in Figure 5c, the device has good moisture adsorption and desorption capacity. When it is exposed to moisture, the device generates an output voltage of 61.48 mV within 2.413 s (stage i). The voltage decreases to 0 mV within 11.18 s (stage ii). The rapid response and recovery originate from oxygen-containing functional groups (-OH, -COOH) on the GO surface, interacting with moisture to establish an interconnected hydrogen-bonded network. This structure contributes to the rapid migration of protons along the ordered pathways, thereby enhancing proton transport efficiency through the formed hydrogen-bonded network. Furthermore, we investigated the different influences of humidity on the device’s output performance. As shown in Figure 5d, the generated output voltage increases with humidity from about 15 mV at 50% to 50 mV at 90%, revealing a pronounced positive correlation between the device’s output voltage and relative humidity levels. Meanwhile, the device on a PET substrate possesses excellent flexibility and mechanical durability. As shown in Figure 5f, the device is bent at different angles of 0°, 90°, and 180°, and the output voltage has no appreciable attenuation under different bending angles. Additionally, the output voltage remains stable over dozens of cycles (Figure 5e). This flexible configuration enables conformal adhesion to irregular surfaces, which overcome the limitations inherent in conventional rigid devices and demonstrate significant advantages for wearable sensing applications.

Large-scale MEGs take advantage of improving and adjusting the output voltage. Benefitting from printing methods and simple drop-coating techniques, we can achieve the interconnection of multiple MEGs (Figure 5g). The scalable units adopt a serpentine connection method, which means the upper electrode of each power generation unit is partially overlapped with the lower electrode of the next unit. The number and size of array units depends on the number of printed lower electrodes, which allows for adjustable configurations according to application requirements. The ten MEGs united as an array are connected as shown in Figure 5h, and the array continuously generates an output voltage exceeding 180 mV under 40% RH. Additionally, exposed to a 70% RH environment, the system exhibits a characteristic voltage spike of about 46.675 mV, and with an increasing of cycle numbers, the output remains stable (Figure 5i). This behavior demonstrates the dual functionality of sustained baseline voltage maintenance and transient spike generation during humidity transitions, confirming the system’s capability for both steady-state power delivery and dynamic humidity variation detection through its transient electrical response characteristics.

### 3.5. Mechanism Analysis

#### 3.5.1. Power Generation Mechanism of MEGs

**Figure 6:**
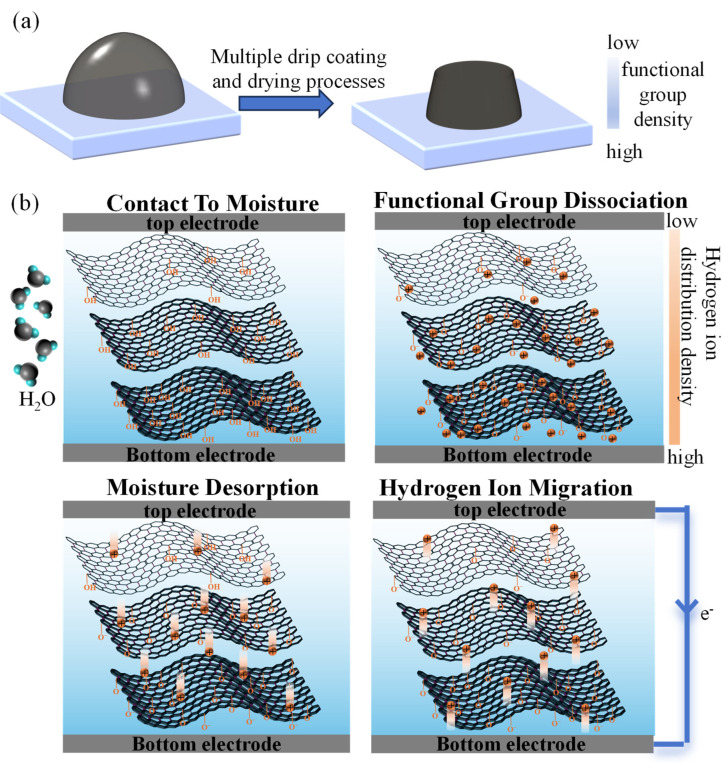
Power generation mechanism of MEG. (**a**) Diagrammatic illustration of droplet morphology; (**b**) diagrammatic illustration of power generation mechanism.

Due to the drop-coating fabrication method used for the GO functional layer, the droplet initially exhibits a wetting behavior on the PET substrate, forming a hemispherical morphology as shown in Figure 6a. During the solvent evaporation process, GO nanosheets tend to deposit toward the substrate interface under the influence of gravity. With repeated droplet deposition, this gravity-driven assembly continues, ultimately resulting in a truncated conical architecture characterized by nanosheet accumulation at the bottom and sparse distribution at the top. This vertical arrangement generates a gradient in oxygen-containing functional group density across the film thickness, with a higher concentration near the bottom and a lower one toward the top. Such a configuration facilitates the formation of an ion concentration gradient when exposed to moisture, thereby enabling voltage generation. The working mechanism of power generation is schematically illustrated in Figure 6b and consists of four sequential stages. First, upon exposure to moisture, the thin GO film rapidly absorbs water molecules. The carboxyl (-COOH) groups then release protons, leaving behind immobilized anionic groups (-COO⁻) on the GO basal planes. Subsequently, due to the asymmetric distribution of GO, a vertical gradient of functional group density is established from top to bottom. This gradient drives the directional migration of free protons, while the fixed anionic groups generate an internal electric field. The movement of these ions induces electron flow in the external circuit, leading to a voltage peak when the proton concentration reaches its maximum. Upon transitioning to a dry environment, the GO film releases absorbed water molecules, causing a decrease in the number of mobile protons. The induced electric field then drives the remaining protons back toward the regions of higher concentration, where they recombine with the negatively charged groups, resulting in voltage decay. This process is fully reversible, allowing the device to return to its initial state and repeat the cycle upon renewed exposure to moisture.

#### 3.5.2. Investigating the Power Generation Effect of rGO/Ag Transition Materials

**Figure 7:**
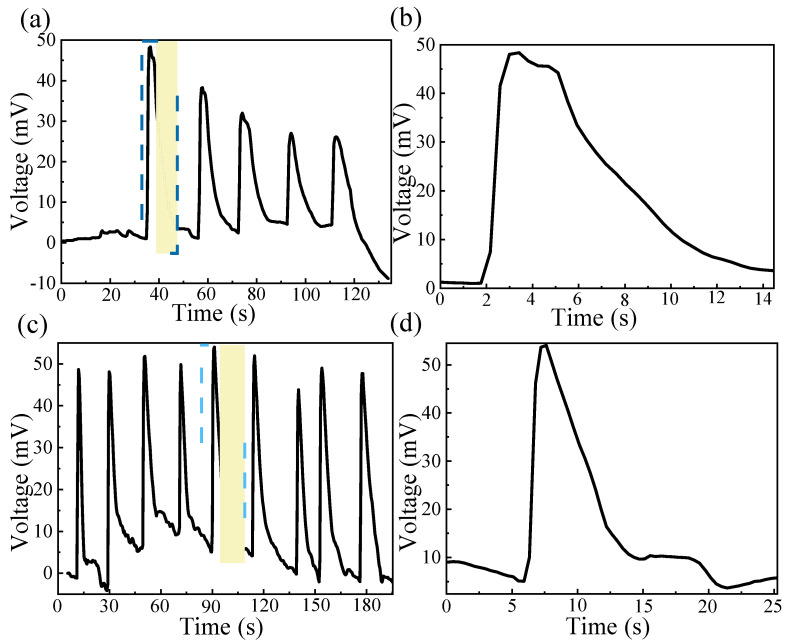
The effect of rGO/Ag transition materials. (**a**) Output voltage cycles of a single MEG without rGO/Ag; (**b**) voltage output of the MEG without rGO/Ag; (**c**) output voltage cycles of a single GO-rGO/Ag MEG; (**d**) voltage output of the GO-rGO/Ag MEG.

To further investigate the role of the rGO/Ag transition layer, a comparative device was fabricated using only a GO active layer without the rGO/Ag component. The devices were tested under a relative humidity (RH) of 95%. As shown in Figure 7a, the device without rGO/Ag exhibited a gradual decrease in output voltage over repeated cycles, dropping from 47.33 mV in the first cycle to 21.60 mV by the fifth cycle. This indicates a progressive decline in humidity sensitivity. The GO-only device achieved a maximum output voltage of 47.33 mV, with a humidity response time of 1.645 s and a recovery time of 8.807 s (Figure 7b). In contrast, the device incorporating the rGO/Ag transition layer maintained a stable output voltage across multiple cycles under sustained 95% RH conditions (Figure 7c). The GO–rGO/Ag device exhibited a maximum output voltage of 48.9 mV, a response time of 1.698 s, and a significantly shorter recovery time of 6.693 s (Figure 7d). These results demonstrate that the presence of the rGO/Ag transition layer effectively stabilizes humidity responsiveness and enhances output consistency during repeated humidity cycling. Furthermore, compared to the device without rGO/Ag, the GO–rGO/Ag device exhibits a reduced recovery time and improved voltage output, thereby enabling faster and more reliable responses to successive humidity changes.

## 4. Conclusions

In summary, we developed a high-performance flexible MEG through the synthesis of GO with elevated hydroxyl group content as a sensing layer and rGO/Ag as a transition layer. The incorporation of rGO/Ag as the transition layer significantly enhanced the device’s cyclic stability, enabling sustained output voltages of 60 mV at a relative humidity of 80%. Through an innovative utilization of gravitational sedimentation effects in drop-casting processes, we achieved vertically functional group gradient distributions that revealed a proton gradient-driven electricity generation mechanism. This breakthrough facilitated the successful fabrication of flexible device arrays demonstrating a dual functionality of sustained hundred-millivolt-range power output and real-time humidity sensing capabilities. This work demonstrates the feasibility of flexible, scalable, and printable fabrication of MEGs, highlighting their potential for wearable electronics, respiratory monitoring systems, and IoT applications. Future implementations could include ultra-thin MEG-integrated breath sensors or smart baby diapers, establishing a broad reference for the practical application of MEGs. While our work establishes a novel paradigm for self-powered system development, particularly in wearable electronics and environmental monitoring applications requiring stable energy harvesting from ambient humidity variations, the limited output voltage of current MEG configurations remains a challenge. Future studies will focus on optimizing material configurations to enhance electrical output.

## Figures and Tables

**Table 1:** Selection of sample preparation process conditions.

Types of GO	Mixed Speed (rpm)	The Amount of KMnO_4_ (g)
GO-1	150	3
GO-2	500	3
GO-3	500	6

**Table 2:** Relative atomic percent composition and C/O ratio of GO and rGO/Ag.

Sample	Atomic Ratio (%)				O1 Element (%)	C/O
	C-C C=C	C-O	C=O	COO		
GO	51.57	35.66	9.33	3.28	30.08	2.3
rGO/Ag	53.87	37.56	8.57	—	11.09	7.56

**Table 3:** Relative atomic percent composition and C/O ratio of GO at different oxidation degrees.

Sample	Atomic Ratio (%)				O1 Element (%)	C/O Ratio
	C-C C=C	C-O	C=O	COO		
GO-1	49.18	28.29	16.2	4.33	30.02	2.33
GO-2	47.82	34.84	11.72	5.56	31.81	2.14
GO-3	49.45	12.58	34.28	3.69	36.26	1.76

## Data Availability

The original contributions presented in this study are included in the article and Supplementary Materials. Further inquiries can be directed to the corresponding authors.

## Supplementary Materials

The following supporting information can be downloaded at https://www.mdpi.com/article/10.3390/ma18122766/s1, Figure S1: The microstructure of GO; Figure S2: XPS survey spectra of rGO/Ag; Figure S3: The SEM image of GO in different oxidation degree; Figure S4: Digital photo of experimental setup; Table S1: Relative atomic percent composition; Table S2: Relative atomic percent composition using the O1s XPS regions deconvolution of GO sample.
