# Supplementary material for: Flexible Moisture–Electric Generator Based on Vertically Graded GO–rGO/Ag Films
Source: Materials (Basel). 2025 Jun 12;18(12):2766. doi: 10.3390/ma18122766 (PMC12194753; doi:10.3390/ma18122766)
Supplement: Supplementary file 1 [file materials-18-02766-s001.zip › materials-3635030-supplementary.pdf]

Flexible Moisture-Electric Generator Based on Vertically Graded GO–rGO/Ag Films

Shujun Wang, Leng Li, Jiayue Wen, Jiayun Feng, He Zhang, Yanhong Tian

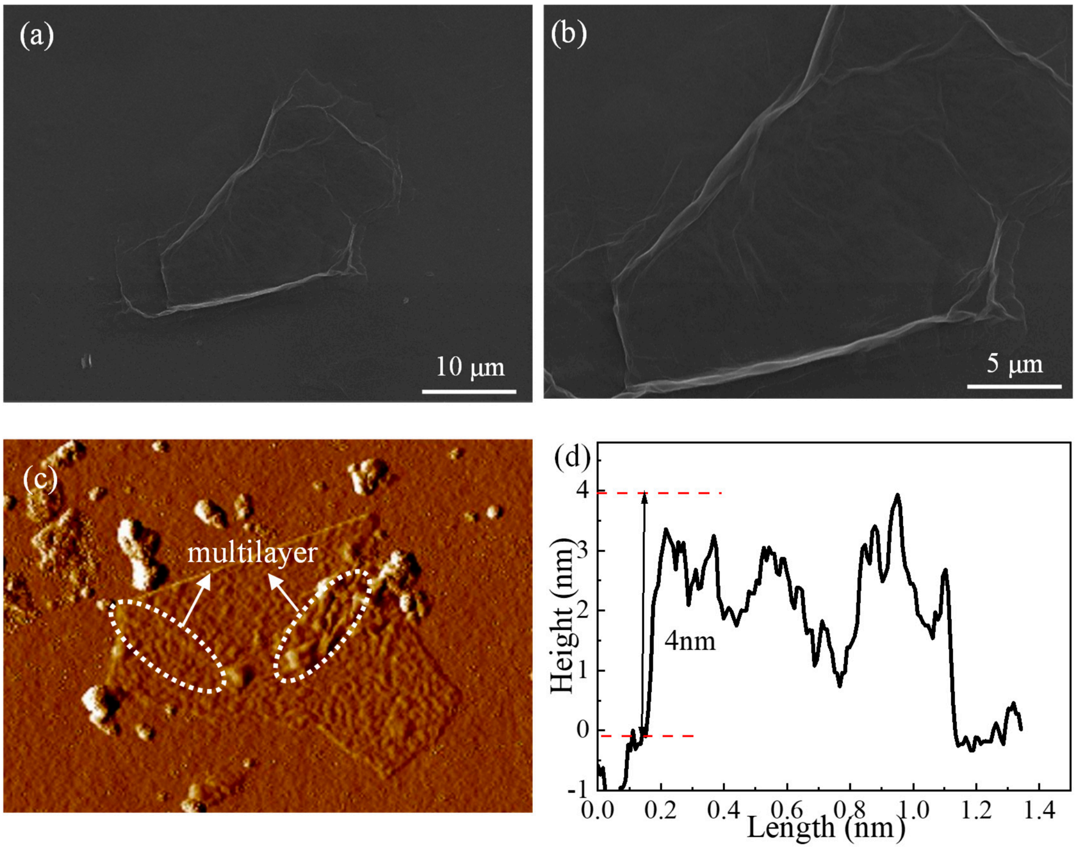

Figure S1 The microstructure of GO

(a)SEM Image at 10 μm scale;(b) SEM Image at 2 μm scale;(c) AFM image;(d)The height of GO

Table S1 Relative atomic percent composition

	C-C/C=C	C-O	C=O	COO
GO	51.74%	35.66%	9.33%	3.28%

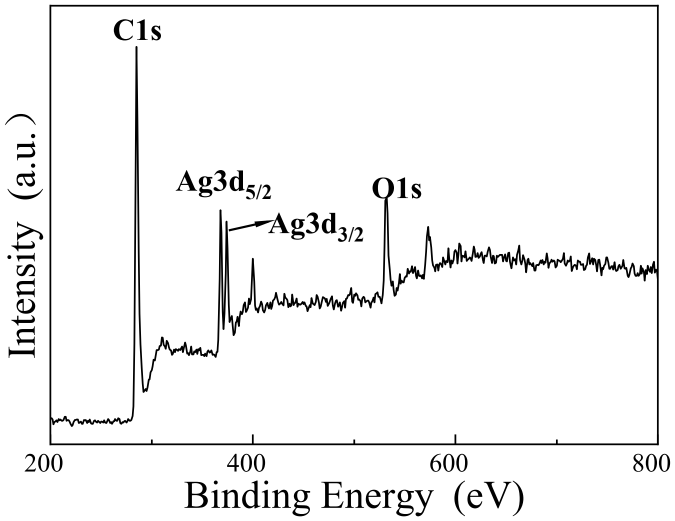

Figure S2 XPS survey spectra of rGO/Ag

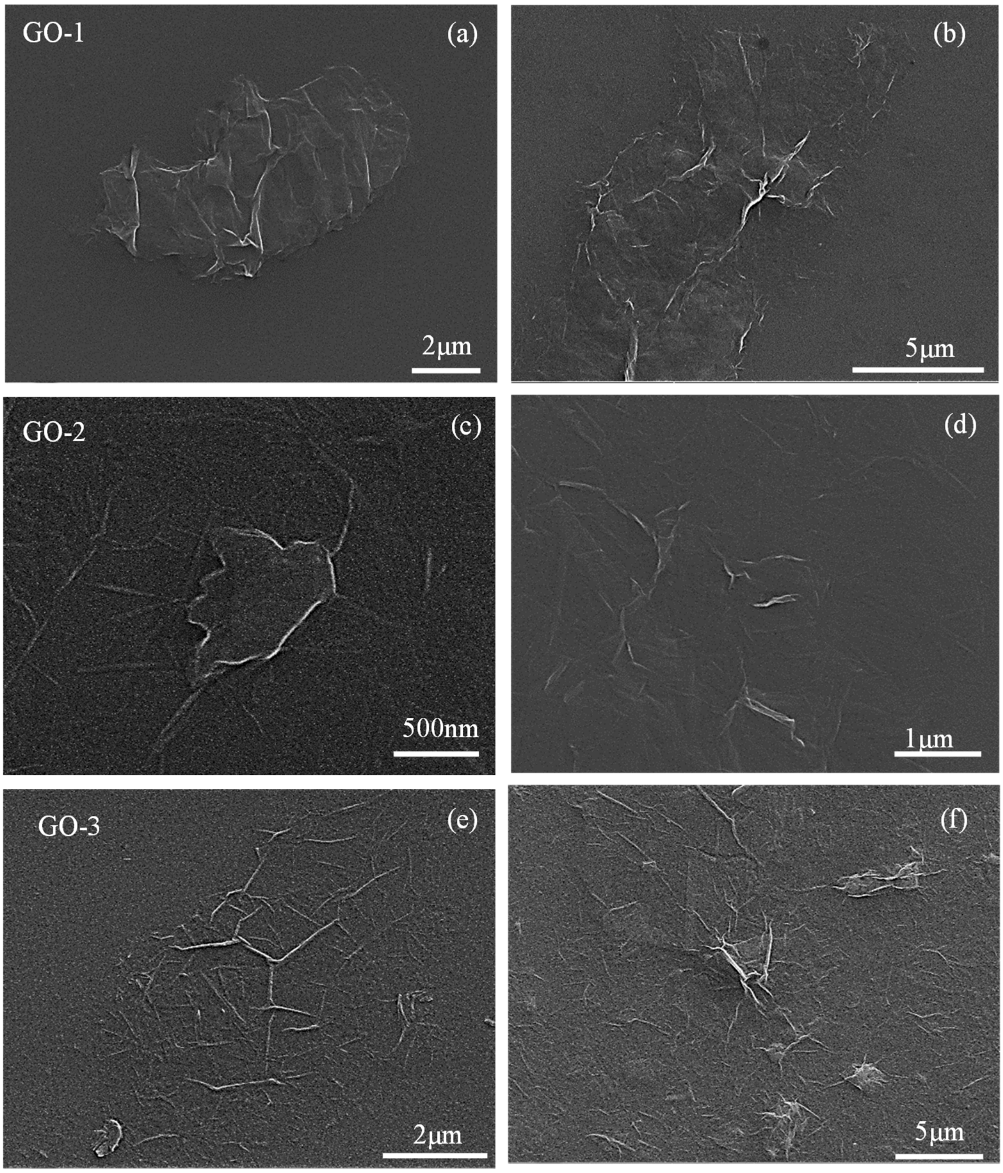

Figure S3 The SEM image of GO in different oxidation degree. (a) SEM image of GO-1 at 2 μm scale; (b) SEM image of GO-1 at 5 μm scale;(c) SEM image of GO-2 at 500 nm scale;(d) SEM image of GO-2 at 1 μm scale;(e) SEM image of GO-3 at 2 μm scale;(f) SEM image of GO-3 at 5 μm scale

Table S2 Relative atomic percent composition using the O1s XPS regions deconvolution of GO sample.

sample	Atomic ratio (%)			
	COOH	C=O	C-O-C	C-OH
GO-1	12.02	——	72.7	15.28
GO-2	9.53	20.47	30.75	39.24
GO-3	9.27	30.25	60.38	——

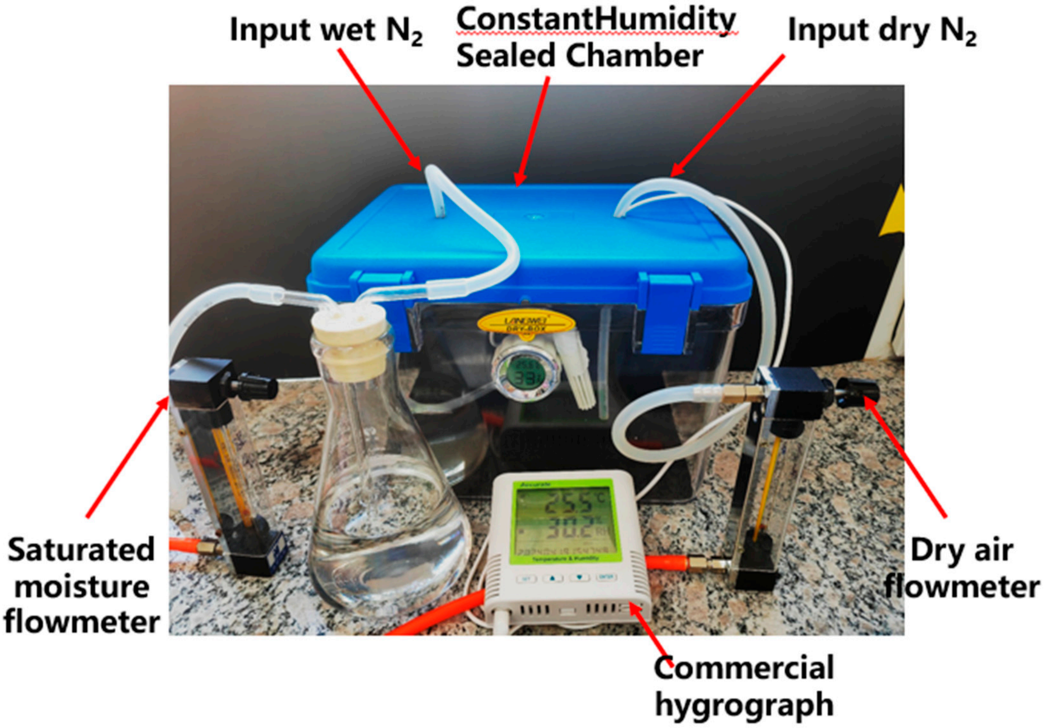

Figure S4 Digital photo of experimental setup
